# Endothelial Protection and Improved Micro- and Macrocirculation with Hemoadsorption in Critically Ill Patients

**DOI:** 10.3390/jcm13237044

**Published:** 2024-11-22

**Authors:** Marton Papp, Can Ince, Jan Bakker, Zsolt Molnar

**Affiliations:** 1 Department of Anesthesiology and Intensive Therapy, Semmelweis University, 78 Üllői St., 1082 Budapest, Hungary; manolo87@gmail.com; 2 Centre for Translational Medicine, Semmelweis University, 22 Baross St., 1085 Budapest, Hungary; 3 Laboratory for Translational Intensive Care, Department of Intensive Care, Erasmus MC, University Medical Centre Rotterdam, 3015 GD Rotterdam, The Netherlands; c.ince@erasmusmc.nl; 4 Department of Pulmonary and Critical Care, New York University School of Medicine, New York, NY 10016, USA; jan.bakker@erasmusmc.nl; 5 Division of Pulmonary, Allergy, and Critical Care Medicine, Columbia University College of Physicians & Surgeons, New York, NY 10032, USA; 6 Department of Intensive Care Adults, Erasmus MC University Medical Center, 3000 GD Rotterdam, The Netherlands; 7 Department of Intensive Care, Pontificia Universidad Católica de Chile, Santiago 8320165, Chile; 8 Department of Anesthesiology and Intensive Therapy and Pain Medicine, Poznan University for Medical Sciences, 49 Przybyszewskiego St, 60-355 Poznan, Poland

**Keywords:** sepsis, septic shock, hemoadsorption, cytokines, endothelium, capillary leakage

## Abstract

A dysregulated immune response is associated with an excessive release of cytokines that can lead to systemic vasoplegia and vasoplegic shock with the development of multiorgan failure that is associated with an increased risk of dying. Under physiological circumstances, the endothelium and the glycocalyx are responsible for maintaining vascular tone, capillary permeability, and hemostasis, and controlling inflammation. In hyperinflammation, the endothelium and glycocalyx become damaged due to the excessive production of certain toxic proteins, along with an overwhelming release of cytokines. It has been shown in both in vitro animal experiments and in humans that extracorporeal hemoadsorption can reduce circulating levels of cytokines and may also remove toxic proteins that directly take part in endothelium and glycocalyx damage. The current review aims to summarize current knowledge, put recent findings into context, and introduce the hypothesis of “endothelial protection with hemoadsorption” in critically ill patients.

## 1. Introduction

A dysregulated host immune response is the primary cause of severe organ dysfunction in hyperinflammatory conditions regardless of the insult, be it of infectious (i.e., sepsis) or non-infectious (i.e., major surgery, trauma, etc.) origin [1]. In 2017, there were nearly 50 million sepsis cases worldwide with an estimated 11 million sepsis-related deaths, which represented almost 20% of all global deaths [2].

As the body cannot control the overwhelming inflammatory response during the early course of the disease, extracorporeal blood purification in the form of hemoadsorption (HA) as an adjuvant therapy has been considered for almost two decades [3].

There are several HA devices available on the market, some of them providing non-selective removal of pro- and anti-inflammatory cytokines and endotoxins (oXiris, Toraymyxin), or even additional adsorption of myoglobin, toxins, and various therapeutic drugs (CytoSorb, HA-330 and HA-380 (Jafron)), selective removal of C-reactive protein (Pentrasorb) or a wide range of bacteria and viruses (Seraph), or selective removal of endotoxin (Efferon) [4,5]. From these, the majority of evidence has been accumulated with the use of CytoSorb, with more than two hundred thousand treatments over the last 10 years (www.cytosorbents.com, accessed on 13 November 2024).

Besides hemoadsorption, there are other potentially beneficial extracorporeal therapies and cytokine-targeted interventions which share the pathophysiological background of HA therapy. For example, plasma exchange as an adjuvant therapy in sepsis and septic shock has been extensively studied in recent decades, but this and other therapeutic modalities are beyond the scope of this review.

Experimental animal data [6,7,8], a recent human study in healthy volunteers [9], and clinical studies in critically ill adults [10,11] and children [12,13] have shown that HA can effectively remove circulating cytokines, and hence could attenuate the dysregulated inflammatory response. Several clinical studies have reported rapid hemodynamic stabilization during HA, as indicated by a significant reduction in the need for vasopressor support and improved cardiovascular variables [14,15,16,17,18,19,20,21]. However, the exact pathophysiological link between reduced cytokine levels and hemodynamic improvement remains unclear. The lack of well-designed studies investigating extracorporeal blood purification is reflected in the actual Surviving Sepsis Guidelines, which state “there is insufficient evidence to make a recommendation on the use of other blood purification techniques” [22].

Nevertheless, two of the latest meta-analyses found that cytokine adsorption may not have a significant effect on circulating cytokine levels (including IL-6) and may be associated with increased mortality in a subset of patients [23,24]. However, we should interpret these results with caution because the confidence in the evidence was low or very low in both studies. There could be several explanations for the potential harmful effects of hemoadsorption therapy in critically ill patients. Firstly, we do not know if non-selective, albeit concentration-dependent, removal of cytokines results in a beneficial or detrimental imbalance of these substances. Secondly, the in vivo cytokine removal ability of these devices is unknown, besides promising in vitro results, which can be explained by several factors. Interleukins are produced continuously and can also be found in the interstitium, and they can migrate to the vasculature after removal. The device’s adsorption capacity decreases rapidly after saturation; therefore, changing intervals can highly influence the performance of the system. Thirdly, CytoSorb can adsorb several drugs, such as antibiotics and antivirals, which may result in inappropriate dosing without correct drug level monitoring [25]. Obviously, this could have negative effects on patient outcomes. Fourthly, HA is mostly used by being connected in series to an extracorporeal circuit, and these artificial surfaces have the potential to cause systemic inflammation, therefore increasing the levels of cytokines in question.

The potential therapeutic effect of cytokine removal could be hypothesized in several so-called hyperinflammatory syndromes, like sepsis, acute respiratory distress syndrome (ARDS), on-pump cardiac surgery, pancreatitis, liver failure, and burns. Most of the clinical studies in question investigate patients with sepsis (mostly septic shock), severe ARDS, or patients undergoing complex cardiac surgery. Under these circumstances, cytokine adsorption happens whilst connected in series with an extracorporeal circuit—cardiopulmonary bypass, ECMO, or continuous renal replacement therapy (CRRT). However, standalone therapy is also feasible. Therefore, hemoadsorption itself does not require extra anticoagulation as the extracorporeal system (with citrate in the case of CRRT) or the patient (with heparin in the case of ECMO) is anticoagulated anyway. Use of the CytoSorb device is not associated with significant removal of coagulation factors, or activation of the complement or coagulation system [26], and only causes a slight and transient decrease in the platelet count. Therefore, the overall effect on the coagulation system is negligible, without the need for fibrinolytic treatment.

The endothelium is a major determinant of circulatory function, platelet aggregation, and vascular tone [27]. Therefore, the current review aims to summarize, contextualize recent findings on, and introduce the hypothesis of “endothelial protection with HA” in critically ill patients.

## 2. The Endothelium and the Glycocalyx

The endothelium and its inner layer, the glycocalyx, are responsible for several essential physiological functions in maintaining homeostasis and adequate end-organ function. The glycocalyx is a web of membrane-bound glycoproteins and proteoglycans on the luminal side of the endothelium. These two structures act in concert to determine a number of vital functions for the circulatory system, including vascular permeability [28], coagulation, inflammation—by preventing leukocytes and platelets from adhering to the vessel walls [29]—and vascular tone, along with vascular smooth muscle [30].

**Figure 1:**
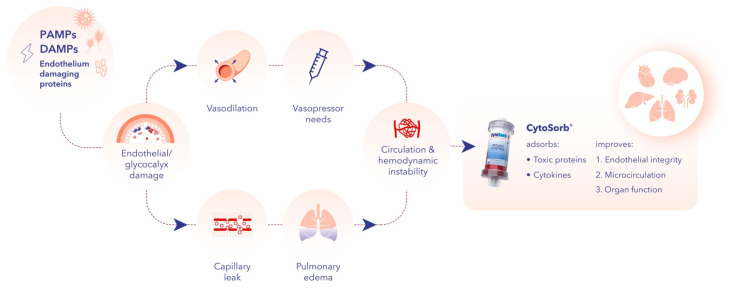
Hyperinflammation causes endothelial damage and protection with hemoadsorption. Legend: PAMPs, pathogen-associated molecular patterns; DAMP, damage-associated molecular patterns. See explanation in text.

It has been shown that this function can be affected by several inflammatory conditions, including sepsis [31]. During hyperinflammation, the endothelium, its connecting adherents, and the glycocalyx become damaged due to the excessive production of inflammatory mediators, along with the overwhelming release of cytokines leading to increased capillary leakage, interstitial edema, and immuno-thrombosis that further amplifies the underlying hyperinflammation and the associated systemic vasoplegia [27,31]. This combination of increased capillary permeability and vasoplegia can be responsible for an absolute (into the tissues) and relative (into the dilated vessels) loss of circulating blood volume that explains the rationale and vital importance of early, adequate fluid resuscitation and vasopressor support (Figure 1).

Resuscitation should begin immediately in septic shock to enhance hemodynamic stabilization, which is stated to be the “Best Practice” by the most recent Surviving Sepsis Campaign Guidelines [22]. Even though fluids and vasopressors are the advised main resuscitation procedures, they do not reduce the inflammatory process themselves. In fact, fluids can promote further amplification of the inflammatory response [32]. Therefore, attenuating the overwhelming inflammatory response following sepsis by extracorporeal removal of inflammatory mediators that can cause damage to the endothelium may mitigate endothelial dysfunction leading to organ failure.

## 3. Endothelium Protection with HA

Hemodynamic instability is the most important manifestation of vasoplegic shock, leading to inadequate microcirculatory perfusion, tissue oxygen deficit, and organ dysfunction. Therefore, maintaining adequate microcirculation should be a primary goal of any therapeutic measure employed to improve hemodynamic status [33]. Recent studies have shown that there is an array of cellular processes that contribute to endothelial damage during the course of septic shock [31,34].

One of the first animal models on this subject tested the efficacy of cytokine adsorption on coronary endothelial function in the setting of a “donation after cardiac death” scenario [35]. Healthy pig hearts were harvested after circulatory death and randomized into three groups: (1) controls, (2) four-hour hemoperfusion without HA, and (3) four-hour hemoperfusion with HA. Before microvascular assessments were performed, all hearts underwent 1 h of reperfusion, and then laser Doppler perfusion (LDP) was performed, and arteriolar perfusates were analyzed. In addition to 13 cytokines, the levels of markers of endothelial oxidative stress (nitrotyrosine and hydroxy-nonenal) as well as endothelial-cell-injury-indicating adhesion molecules (CD54, CD106, CD31) were reduced, and LDP improved in the HA group. The authors concluded that HA prevented oxidative stress and ischemia–reperfusion injury of the endothelium in the coronary arterioles. Unfortunately, the data were insufficient to provide a clear explanation of the results.

A very similar approach was applied in two further in vitro experiments. In both studies, the authors retrieved HA cartridges after their use in hyperinflamed patients and washed out the cellular elements of blood components and non-adsorbed plasma proteins. Adsorbed proteins were then detached by rinsing the cartridges with acetonitrile. The proteins were identified with high-performance liquid chromatography and lyophilized, and then resuspended to form different elutions. Endothelial cell cultures were then treated with this elution, and measurements were performed. This way, Denzinger et al. [36] were able to identify 39 protein fractions, of which 3 fractions had a detrimental effect on the endothelial cells by inhibiting cell proliferation, increasing cell death, and inducing cell apoptosis. Piskovatska et al. [37] found similar results and also identified concentration-dependent harm. They showed that the majority (83; 65.4%) of the isolated proteins were in the molecular weight range below 60 kDa and reported that the higher the protein concentration in the elute, the lower the percentage of viable cells in the culture. In fact, at the highest concentration, cell death was similar to that of cells treated with highly toxic hydrogen peroxide (H_2_O_2_). The authors also investigated the effects of these proteins on “wound healing” by scarring the endothelial cell culture and evaluating regeneration (i.e., healing). In the control cultures, scars disappeared within 24 h, while the addition of the elute, even at the lowest concentration, prevented endothelial regeneration. The authors concluded that HA allowed for broad-spectrum removal of a wide range of molecules that cause endothelial damage.

David et al. [38] treated human umbilical vein endothelial cells (HUVECs) in vitro with serum obtained from a septic shock patient receiving HA therapy. Serum samples were collected before and after 24 h of HA, and their effects on the HUVECs were investigated and then compared to treating HUVECs with serum from a healthy control. Morphological changes were measured by immunofluorescent immunochemistry, and functional changes (i.e., capillary permeability) were measured with transendothelial resistance (TER). Treatment with the serum obtained before HA resulted in a substantial disruption of the endothelial cellular junctions and a drop in TER compared to controls. When HUVECs were treated with the serum collected after HA therapy, disruption did not take place, and TER remained significantly higher. The authors suggested that HA might have cleared substances responsible for the loss of endothelial integrity caused by the septic serum. However, one cannot exclude that the results were due to the natural course of the disease, i.e., the collected blood at time zero behaved differently from the blood collected 24 h later, and changes were not necessarily the direct effect of HA. Nevertheless, the results are intriguing and render further clinical studies necessary.

## 4. Other Potential Inflammatory Markers That Could Damage the Glycocalyx

Several inflammatory markers are routinely measured in everyday clinical practice to aid decision making in diagnosis and treatment. Most of these have been shown to be effectively adsorbed by HA.

In a recent clinical experimental study, Drost et al. found that interleukin—IL-6—was strongly associated with glycocalyx damage and correlated both with glycocalyx dimensions and circulating biomarkers [39]. Exposure of endothelial cells to 5% serum from COVID-19 or sepsis patients resulted in a significant decrease in glycocalyx height, which was attenuated by co-incubation with tocilizumab. In 219 COVID-19 patients, a previously identified proteomic glycocalyx signature correlated with IL-6 (*p* < 0.0001). These data suggest that IL-6 may significantly drive endothelial damage in COVID-19 and bacterial sepsis. A recent observation in human volunteers found that HA effectively removes IL-6 (−71%, *p* = 0.003 was observed in the CytoSorb group) [9]. Therefore, it is a logical assumption that reducing circulating levels of IL-6 could have some effects on endothelial protection.

One of the most frequently used and investigated inflammatory biomarkers is procalcitonin (PCT). Although PCT has routinely been used as a diagnostic inflammatory marker worldwide for 25 years, it has also been suggested to mediate the underlying disease process [40]. In their study, Nylen et al. used an *E. coli* peritonitis model in hamsters and administered exogenous PCT to one group of septic animals, which significantly increased mortality compared to non-treated septic animals (93% vs. 43%, *p* = 0.02). On the other hand, pretreating the animals with PCT antiserum almost completely prevented mortality compared to the septic controls (6% vs. 62%, *p* = 0.003). A recent observational clinical study combined with in vitro and in vivo experiments provides further strong support to the hypothesis that PCT, in fact, has a direct damaging effect on the endothelium [41]. The authors found that, on the one hand, elevated PCT levels were associated with microvascular dysfunction and increased capillary leakage in patients undergoing cardiac surgery, as indicated by a 1.4-fold increase in endothelial and 2.3-fold increase in pulmonary capillary permeability (both *p* values = 0.001). Furthermore, the authors’ in vitro and in vivo experiments provided evidence that these effects were due to the direct effects of PCT on the endothelium by destabilizing VE-cadherin and also that inhibiting PCT’s action with olcegepant reduced capillary leakage. Available data indicate that HA can rapidly and significantly decrease circulating PCT concentrations [15,16,42,43], supporting the hypothesis that reducing PCT levels with HA may inhibit the action of PCT on the endothelium, which could help to preserve vascular integrity.

Another potential biomarker of interest is the soluble urokinase-type plasminogen activator receptor (suPAR) [44]. This molecule, expressed in various immune cell membranes, is associated with innate and adaptive immunity: endothelial cells, macrophages, neutrophils, and activated T-cells [45]. It is released into body fluids during inflammatory stimulation and has been associated with endothelial dysfunction, especially in patients with nephrotic syndrome [46,47]. Roca et al., in an observational cross-sectional study in patients with nephrotic syndrome, reported a significant correlation between suPAR and vascular cell adhesion molecule-1 (VCAM-1) and syndecan-1, two established biomarkers of endothelial and glycocalyx injury [46]. The results of a more recent study by Nusshag et al. not only support the relevance of suPAR as a new diagnostic biomarker but, more importantly, strengthen the concept that suPAR is a direct pathophysiological driver involved in sepsis-induced AKI (patients with suPAR levels of greater than 12.7 ng/mL were at highest risk for RRT or death, with an adjusted odds ratio of 7.48 (95% CI, 3.00–18.63) [47]. Furthermore, in this polymicrobial murine sepsis model, they found pronounced ultrastructural kidney damage, impaired kidney function, and poor survival associated with high blood sugar levels. In a case series of COVID-19 patients and in a kidney transplant patient it was shown that suPAR can be adsorbed by HA [45,48], also indicating a potential protective role of HA in this context.

The third potential biomarker exhibiting mediator properties is high mobility group box-1 (HMGB-1). HMGB-1, circulating histones, and glycans such as heparan sulfate are considered damage-associated molecular patterns (DAMPs) [49]. Among them, HMGB-1 and histones have been extensively studied because they significantly mediate lethal systemic inflammation, complement and coagulation activation, endothelial injury, and organ dysfunction in various critical illnesses, such as sepsis [50], acute liver failure [51], pancreatitis [52], multiple trauma [53], and severe COVID-19 [54]. Effective adsorption of HMGB-1 by HA has recently been shown by Gruda et al. (83–98% removal during a 5 h HA) [55]. Subsequently, Weber et al. found that 6 hrs HA significantly decreased circulating histone levels in the blood of 22 humans with multiple injuries (depending on different histone concentrations, the decrease was between 92% and 99%) [56].

In summary, these early mechanistic data look promising and can help inform the design of future studies investigating the potential of active mediator removal by HA as a novel approach to protect endothelial integrity and contribute to circulatory recovery in critically ill patients. It is also important to note that although there are several HA devices on the market, all of the preclinical and clinical studies on this topic to date used CytoSorb (CytoSorbents Corporation, Monmouth Junction, NJ, USA); hence, the potential performance of other devices in this regard can only be speculated about, something that should also be considered when future research is planned.

## 5. Clinical Implications

Endothelial protection by HA in critically ill patients could have important clinical implications. Efficient removal of toxic substances that impair endothelial integrity may help sustain endothelial function in support of organ function. Better microcirculatory flow and perfusion could also lead to faster hemodynamic stabilization and improved tissue oxygen uptake. Improved macro- and microcirculation performance with hemodynamic stabilization could also reduce excessive fluid administration and prolonged vasopressor support, which have been associated with unfavorable outcomes [33,57,58].

## 6. Limitations and Future Perspectives

Despite the above detailed promising results, clinical data and well-designed prospective studies are still lacking. There is an urgent need for randomized controlled trials with prespecified standardized hemoadsorption protocols for the investigation of endothelial protection and its impact on patient outcomes. In human case reports and case series, authors report that HA therapy resulted in improved microcirculatory flow, assessed in the sublingual region in septic shock patients that was also associated with hemodynamic stabilization and improvements in metabolic acidosis and overall sequential organ failure assessment (SOFA) scores [11,59]. Such studies suggest that sublingual microcirculatory monitoring may provide a useful diagnostic tool for evaluating endothelial and microcirculatory function to guide the use and timing of HA using microcirculatory improvement as a clinical target for resuscitation [60].

In one of the earliest case reports in a patient treated with severe acute respiratory distress syndrome (ARDS), HA appeared to decrease capillary leakage and alveolar fluid accumulation, as evidenced by improved respiratory function, a significantly reduced need for fluid resuscitation, and achievement of a negative fluid balance shortly after therapy initiation [61]. In a recent multicenter study in critically ill COVID-19 patients with respiratory failure requiring extracorporeal membrane oxygenation (ECMO) support who were also treated with HA, Hayanga et al. reported higher survival rates compared with the global experience in such patients treated with ECMO alone [62]. The results of a recent retrospective study on 124 septic patients suggest that HA therapy is associated with a reduced positive fluid balance and a reduction in vasopressor needs that may, in part, be attributable to potential positive effects on endothelial integrity [63]. Finally, a recent systematic review and meta-analysis found that HA in ARDS patients resulted in a reduction in vasopressor requirements and a decrease in CRP levels as well as a significant improvement in oxygenation, potentially corresponding to improvements in endothelial function and lower fluid balance [64].

## 7. Conclusions

There are robust data showing that hyperinflammation contributes to endothelial damage, which, in turn, triggers the deleterious pathophysiological cascade of lost endothelial function leading to vasoplegia, resulting in tissue hypoperfusion, organ failure, and ultimately death. Hemoadsorption is capable of removing circulating cytokines, inflammatory mediators, and additional toxic proteins responsible for endothelial damage, thereby potentially exerting endothelial protection and improving micro- and macrocirculatory function. Whether these mechanistic effects translate into palpable clinical benefits is a question for future research.
